# A multi-faceted knowledge translation approach to support persons with stroke and cognitive impairment: evaluation protocol

**DOI:** 10.1186/s13012-015-0346-6

**Published:** 2015-11-05

**Authors:** Sara E McEwen, Michelle Donald, Deirdre Dawson, Mary Y Egan, Anne Hunt, Sylvia Quant, Sharron Runions, Elizabeth Linkewich

**Affiliations:** Sunnybrook Research Institute, Toronto, Canada; University of Toronto, Toronto, Canada; Sunnybrook Health Sciences Centre, Toronto, Canada; Rotman Research Institute, Baycrest, Toronto, Canada; University of Ottawa, Ottawa, Canada; Bloorview Research Institute and Holland Bloorview Kids Rehab Hospital, Toronto, Canada

**Keywords:** Cognitive orientation to daily occupational performance, Stroke, Cognitive impairment, Rehabilitation, Knowledge translation, Interrupted time series, Protocol

## Abstract

**Background:**

Patients with cognitive impairments following a stroke are often denied access to inpatient rehabilitation. The few patients with cognitive impairment admitted to rehabilitation generally receive services based on outdated impairment-reduction models, rather than recommended function-based approaches. Both reduced access to rehabilitation and the knowledge-to-practice gap stem from a reported lack of skills and knowledge regarding cognitive rehabilitation on the part of inpatient rehabilitation team members. To address these issues, a multi-faceted knowledge translation (KT) initiative will be implemented and evaluated. It will be targeted specifically at the inter-professional application of the cognitive orientation to daily occupational performance (CO-OP). CO-OP training combined with KT support is called CO-OP KT. The long-term objective of CO-OP KT is to optimize functional outcomes for individuals with stroke and cognitive impairments. Three research questions are posed:

1. Is the implementation of CO-OP KT associated with a change in the proportion of patients with cognitive impairment following a stroke accepted to inpatient rehabilitation?
2. Is the implementation of CO-OP KT associated with a change in rehabilitation clinicians’ practice, knowledge, and self-efficacy related to implementing the CO-OP approach, immediately following and 1 year later?
3. Is CO-OP KT associated with changes in activity, participation, and self-efficacy to perform daily activities in patients with cognitive impairment following stroke at discharge from inpatient rehabilitation and at 1-, 3-, and 6-month follow-ups?

**Methods/Design:**

Three interrelated studies will be conducted. Study 1 will be a quasi-experimental, interrupted time series design measuring monthly summaries of stroke unit level data. Study 2, which relates to changes in health care professional practice and self-efficacy, will be a single group pre-post evaluation design incorporating chart audits and a self-report survey. Study 3 will assess patient functional outcomes using a non-randomized design with historical controls. Assessments will occur during admission and discharge from rehabilitation and at 1, 3, and 6 months following discharge from rehabilitation.

**Discussion:**

This project will advance knowledge about the degree to which the implementation of a supported KT initiative can sustainably change health system, knowledge, and patient outcomes.

## Background

Patients with cognitive impairments following a stroke are often denied access to inpatient rehabilitation [1], despite evidence of its benefits for them [2]. These patients comprise up to 30 % of stroke patients [3]. In the instances when they are admitted to inpatient stroke rehabilitation, they generally receive services based on outdated impairment-reduction models, rather than recommended function-based approaches [4]. These two issues, reduced access to rehabilitation and the knowledge-to-practice gap, both stem from a reported lack of skills and knowledge on the part of stroke rehabilitation teams [4]. To address these issues, we will implement and evaluate a multi-faceted, supported, integrated knowledge translation (KT) initiative, targeted specifically at the inter-professional application of the cognitive orientation to daily occupational performance (CO-OP) approach [5]. The CO-OP approach is a contemporary, effective, cognitive strategy-based treatment approach aligned with Canadian stroke best practice recommendations for cognitive rehabilitation [6]. The long-term objective of this knowledge translation initiative is to optimize functional outcomes for individuals with cognitive impairments following a stroke. Three outputs are expected: (1) increased proportion of patients with cognitive impairments admitted to inpatient stroke rehabilitation; (2) enhanced capacity of inter-professional stroke rehabilitation team members to implement a cognitive-strategy based treatment approach; and (3) improved immediate and long-term functional outcomes for patients with cognitive impairments discharged from inpatient stroke rehabilitation.

This project is a collaboration between a group of knowledge users, lead by Ms. Elizabeth Linkewich, and a group of researchers, lead by Dr. Sara McEwen. Lead knowledge user and decision maker, Ms. Linkewich, is one of three regional directors within the Toronto Stroke Networks (TSNs), an organization charged with overseeing service delivery is in line with best practices and optimization of resources [7]. Ms. Linkewich has convened a knowledge user team of educators, a community re-engagement specialist, and management collaborators. To ensure an integrative KT strategy, these partners have been involved with every aspect of this project’s design, beginning with pre-project work to identify the problem and develop plans to address it.

The Toronto Stroke Networks developed a model to aid discharge from acute stroke care in a way that would maximize both patient outcomes and the efficient use of scarce rehabilitation resources, considering severity and patient characteristics [1]. The model indicates that 40 % of patients discharged from acute care should be transferred to specialized inpatient rehabilitation. However, in recent years, the actual proportion has ranged from 26 to 33 %, and the discrepancy comes in part from patients with cognitive impairment. Lack of access to inpatient rehabilitation may result in patients being transferred to assisted living facilities without the benefit of rehabilitation that could potentially have enabled them to return home. It may also contribute to the relatively poorer outcomes of patients with cognitive impairments, including higher rates of dependency and disability and lower mood and quality of life [8].

Ontario’s Ministry of Health and Long-Term Care and the TSNs have embedded access to inpatient rehabilitation for patients with cognitive impairment into care expectations [9]. However, inter-professional team members’ perceived lack of skills to support patients with cognitive impairment remains a major barrier to access to care. Interviews with professionals from Toronto-based rehabilitation teams revealed two important and related knowledge to practice gaps [4]. First, team members reported that they did not have the specialized knowledge and skills to work with stroke patients who were experiencing cognitive impairment. Second, when treating patients with cognitive impairments, team members reported using impairment-reduction models, considered to be outdated, rather than currently recommended function-based models that incorporate problem solving and strategy training. Contemporary function-based models are substantially better than pure impairment reduction models, in that they are associated with sustained improvements in everyday functioning, self-evaluation, and problem solving, among several other benefits [2]. Canadian Stroke Best Practice Recommendations state that interventions should be tailored to meet meaningful, functional patient goals [6]. The CO-OP approach aligns with these recommendations, and has demonstrated efficacy to improve function in people with stroke [10–12], including those with demonstrated cognitive impairment [12–14].

CO-OP, a functional, patient-goal-centered, problem-solving approach, is associated with improved function, activity performance, participation, and self-efficacy in people with stroke [10, 11, 13], and has demonstrated better efficacy than control conditions [12, 14, 15]. Table 1 provides an overview of relevant CO-OP stroke publications. CO-OP has seven key features that include client-chosen goals, dynamic performance analysis, cognitive strategy use, and guided discovery. In the first session, the patient and a rehabilitation clinician work together, using the Canadian occupational performance measure (COPM) [16], to select personally-valued activities which the patient needs or wants to do. These then form the basis of their rehabilitation goals and become the direct focus of the intervention. In the second session, the patient is taught a global cognitive strategy (Goal-Plan-Do-Check). This strategy is used in an iterative manner in all future sessions as the main problem-solving framework to facilitate skill acquisition/goal attainment. The patient identifies a goal, is guided by the clinician to discover a plan to achieve the goal, does the plan, and finally checks to see if the plan was implemented and if it worked to achieve the goal. Within the plan phase, the clinician uses guided discovery rather than explicit instruction to help the patient analyze the task to be performed and to discover individual strategies that are specific to the particular performance problems of that patient with that chosen activity. A detailed description of CO-OP’s theoretical foundations, key features, and administration procedures is available in a publication by Polatajko and Mandich, 2004 [5].

CO-OP has an established training infrastructure with available local trainers, and therefore is feasible and efficient to implement. However, given the multi-institutional environment, the need for shifts in attitudes and beliefs (the CO-OP approach requires therapists to give up a significant degree of control to the clients) and the relative ineffectiveness of passive KT strategies [17], CO-OP training by itself is unlikely to be sufficient to cause widespread, sustained practice change. Thus, we will implement a multi-faceted KT initiative that will combine institution-specific support, multi-sectoral collaboration, and managerial participation along with the established CO-OP training. These additional health system components are important in moving evidence to practice in complex environments, particularly when shifts in culture, attitudes, and behavior are required [18, 19]. Components of the project will integrate seamlessly with existing TSNs’ education and KT infrastructure, and will incorporate online components, including a virtual community of practice. These aspects will facilitate sustainability.

**Fig. 1:**
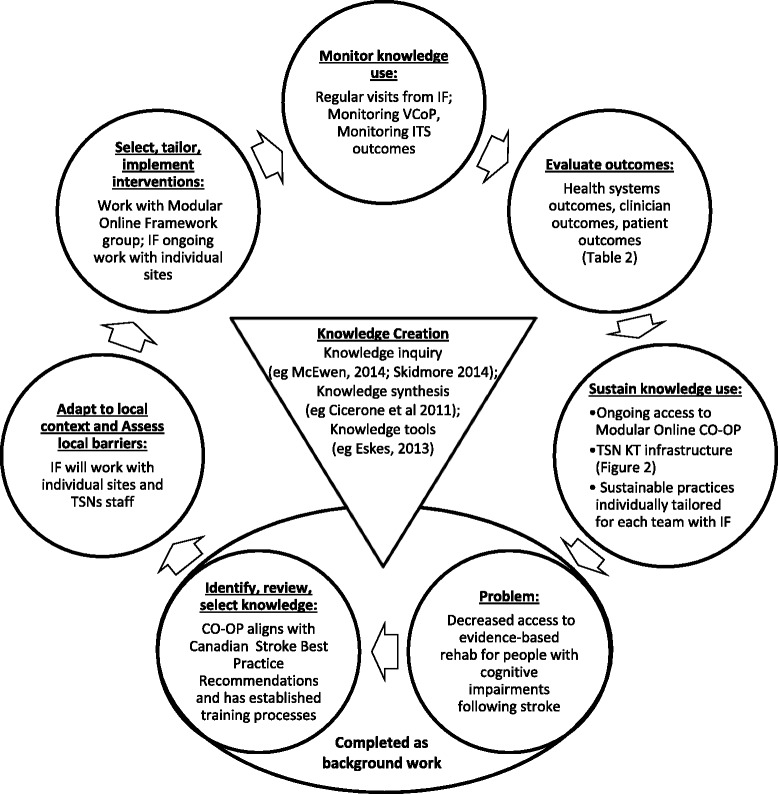
Knowledge to action framework with CO-OP KT project content. Adapted from Graham et al. [20], the KTA framework consists of an inner knowledge creation cycle, depicted here as an *inverse triangle*; and a concurrent action cycle, depicted here as the *external circles. CO*-*OP* cognitive orientation to occupational performance, *IF* implementation facilitator, *TSNs* Toronto Stroke Networks, *VCoP* virtual community of practice, *ITS* interrupted time series, *KT* knowledge translation

The knowledge to action framework developed by Graham et al. provides the foundation for this project [20]. The knowledge to action framework consists of a central knowledge creation cycle and a concurrent action cycle. Figure 1 depicts the knowledge to action framework with content specific to the CO-OP KT project. In preparatory work, issues were identified and knowledge was synthesized to develop strategies to mitigate those issues. The results led to development of the current project which begins with the phase of adapting the knowledge to the local context and identifying barriers to knowledge uptake, and will continue with selecting, tailoring, and implementing KT intervention, monitoring and sustaining knowledge use, and evaluating outcomes.

Also informing the project are the inter-professional collaboration and inter-professional education constructs. Inter-professional collaboration incorporates effective working relationships among health care providers from different disciplines and their patients and enables optimal health outcomes by building on the foundational elements of “respect, trust, shared decision making, and partnerships” [21]. Inter-professional education emphasizes providers from multiple differences learning with, from, and about each other, and is interdependent with inter-professional collaboration.

Through this project, we expect to see improved knowledge and practice change in inter-professional stroke rehabilitation teams, manifested as greater implementation of cognitive rehabilitation best practices and greater self-efficacy to do so. Following directly from positive practice change, we also expect to see improvements at the health system and health outcome levels. Individual rehabilitation professionals and teams as a whole will come to see themselves as having the specialized knowledge and skills to facilitate functional improvement in patients with cognitive impairments. With this, the number of patients with cognitive impairment who are accepted to inpatient rehabilitation will increase. Further, because previous research indicates that CO-OP is associated with improvements in functional independence, activity performance, self-efficacy, and participation, including in those with demonstrated cognitive impairment [10–15, 22, 23], we expect to see similar positive patient outcomes. Aligning with the three expected project outputs outlined previously in the manuscript, three specific research questions are posed:

1. Is the implementation of CO-OP KT associated with a change in the proportion of patients with cognitive impairment following a stroke accepted to inpatient rehabilitation?
2. Is the implementation of CO-OP KT associated with a change in rehabilitation clinicians’ practice, knowledge and self-efficacy related to implementing the CO-OP approach, immediately following and 1 year later?
3. Is CO-OP KT associated with changes in activity, participation, and self-efficacy to perform daily activities in patients with cognitive impairment following stroke at discharge from inpatient rehabilitation and at 1-, 3-, and 6-month follow-ups?

## Methods/Design

**Fig. 2:**
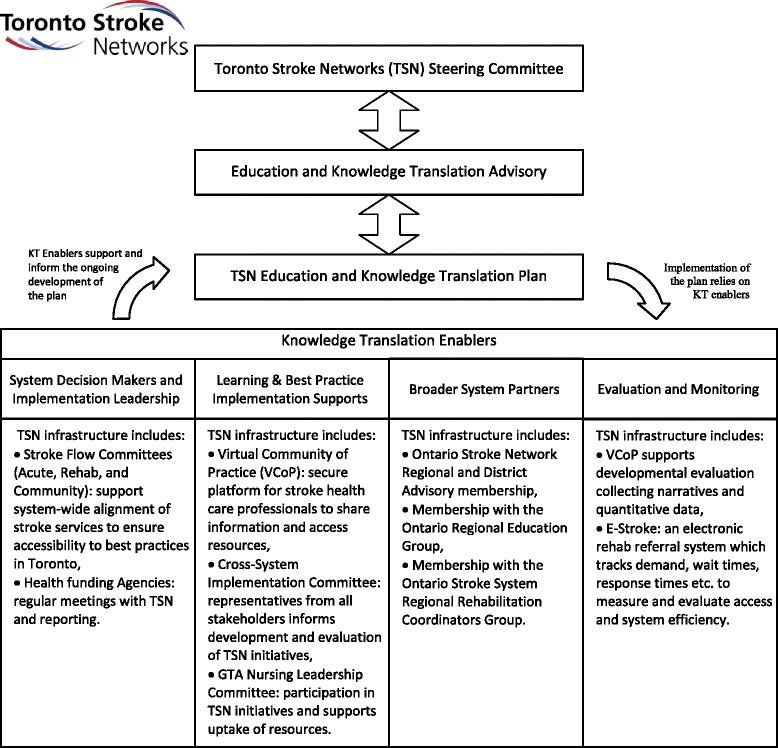
Toronto stroke networks knowledge translation (KT) infrastructure

To answer the three questions, three interrelated studies will be conducted at five inpatient rehabilitation units in the Greater Toronto Area. See Fig. 2 for a visual depiction of all three studies and expected timeline. The first question, which relates to changes at the health system level, will be addressed using a quasi-experimental, interrupted time series design (Study 1); the second, which relates to changes in health-care professional knowledge, practice, and self-efficacy, will be addressed using a single group pre-post evaluation design (Study 2); and the third, which relates to patient outcomes, will be addressed using a non-randomized design with historical controls (Study 3). Table 2 provides an overview of outcomes, indicators, and their timing for all three studies.

### Study 1: admission to rehabilitation

An interrupted time series (ITS) design provides an estimate of the effect of an intervention using a long series of measurements of dependent variables, divided into pre-intervention and post-intervention segments [24]. It is useful when randomized designs are impossible or impractical, as is the case here.

Several years of retrospective rehabilitation referral and admission data are available through the TSNs. Monthly pre-intervention time points will collected for 28 months prior to the intervention implementation period, and post-intervention time points will be collected for 15 months thereafter. Thus, there will be 28 pre-intervention time points, called −T28 (intervention implementation minus 28 months), −T27, etc., to −T1. Following −T1, the CO-OP KT intervention will be implemented over a 3-month period. This 3-month intervention implementation period is called T0, during which, no data collection will occur. Next, 15 post-intervention monthly time points called T1, T2, etc., to T15 will occur. Aggregate electronic data will be collected at all pre- and post-intervention time points from the National Rehabilitation Reporting System (NRS) and the TSN’s E-Stroke rehab referral system. Data elements will include monthly summaries for the total number of rehabilitation referrals, number of rehabilitation admissions, number of referrals declined, reasons for declined referrals, age, sex, functional independence measure (FIM)™ [25] total, motor and cognitive scores of those admitted to rehabilitation, and discharge location.

### Study 2: changes in team members’ practice, knowledge, and self-efficacy

A pre-post study with a single group of stroke rehabilitation clinicians will be conducted. All stroke rehabilitation clinicians from the five participating sites will be invited to participate, and consenting staff will be enrolled. Indicators of practice change, knowledge change, and self-efficacy are described in Table 1. Chart audits will be used to assess practice change [26], knowledge tests prepared by CO-OP trainers will be used to assess knowledge, and a self-report survey will evaluate changes in self-efficacy. These will be administered immediately before the CO-OP KT intervention (−T1), immediately following (T1), and at a 1-year follow-up (T12). To develop an in-depth understanding of team processes, practices, attitudes related to adoption, and sustainability of best practices for cognitive rehabilitation, site-specific focus groups will be held with consenting clinicians before the intervention (−T1) and at the 1-year follow-up (T12).

**Table 1:** Summary of key CO-OP stroke publications

Authors	Year	Population description	Study design, intervention and control	Main findings
McEwen, S., Polatajko, P., Huijbregts, M., Ryan, J.	2009	Adults living in the community with chronic stroke; 3 single case experiments	Single case experiments. Intervention: CO-OP therapy was administered at the rate of one or two sessions per week, up to 10 sessions were completed. Single case paradigm, participants were their own control	-PQRS [10] scores showed significant improvement in at least 2/3 self-selected functional activities, further improvements at 1-month follow-up. Evidence of skill acquisition and retention
McEwen, S., Polatajko, P., Huijbregts, M., Ryan, J.	2010	Adults with chronic stroke living in the community; 3 single case experiments	Single case experiments. Intervention: Up to 10 CO-OP sessions were completed. Single case paradigm, participants were their own control	-PQRS scores improved for all participants at follow-up in all trained and untrained skills, providing evidence of retention and transfer
Skidmore, E., Holm, M., Whyte, E., Dew, M., Dawson, D., Becker, J.	2011	Single case admitted to an inpatient rehabilitation unit, cognitively impaired; Age 31; male; time since stroke 7 days	Single case study. Intervention 10 45-min CO-OP sessions. In addition, patient received usual inpatient rehabilitation	-Mean improvement of 6.1 on COPM [16] Performance scale score
				-Pittsburgh Rehabilitation participation Scale [measure rehab engagement on 6 point scale] scores improved from 3.2 to 4.9
				-FIM™ [25] scores improved from 68 to 97
				-Improvement in self-care skills
Skidmore, E., Dawson, D., Whyte, E., Butters, M., Dew, M., Grattan, E., Becker, J., Holm, M	2014	Cognitively-impaired patients admitted to an inpatient rehabilitation unit; N = 10; mean age: 68; male: 70 %; mean time since stroke: 14.5 days; mean length of stay: 22 days	RCT. Intervention: CO-OP therapy was administered daily in 30–40 min sessions for the duration of length of stay	-CO-OP participants demonstrated less disability than control participants, FIM™ 117 vs 96
			Control: dose-matched sessions using scripted and open-ended questions to promote reflection on rehabilitation activities and experiences	
			Both groups received usual inpatient rehabilitation in addition to the research interventions	
McEwen, S., Polatajko, H., Baum, C., Rios, J., Cirone, D., Doherty, M., Wolf, T.	2014	Patients admitted to an outpatient rehabilitation program; *N* = 26; mean age: 56; male: 62.9 %; mean time since stroke: 43.3 days; mean number of sessions attended: 12.2 (CO-OP), 13.3 (control). Includes patients with cognitive impairment	RCT. Intervention: CO-OP therapy sessions were 45 min long and administered twice per week for a maximum of 10 sessions, instead of usual occupational therapy. More complex patients who required additional treatment received additional usual care OT	-CO-OP demonstrated a large effect over usual care on performance of functional activities (PQRS) on trained and untrained activities at follow-up, providing evidence of retention and transfer
			Control: participants received usual occupational therapy	-CO-OP also demonstrated a medium effect on participation and self-efficacy, compared to usual care

Project team members’ names were italicized

*CO*-*OP* cognitive orientation to daily occupational performance, *PQRS* performance quality rating scale, *COPM* Canadian occupational performance measure, *FIM*™ functional independence measure

**Table 2:** Outcomes, indicators, and timing for all studies

Outcomes	Indicators and description	Timing
Study 1: health system: data obtained from electronic referral system, health record, and NRS		
Access to inpatient rehabilitation	Monthly totals: # of inpatient rehab referrals, # of admissions, # declined; reasons for declined referrals	• −T28 to −T1 • T1 to T15
Inpatient rehab outcomes	Average monthly functional independence measure (FIM™) motor and cognitive scores (admission, discharge, and change)	• −T28 to −T1 • T1 to T15
	Monthly frequency of discharge locations (home, home with services, assisted living facility, or acute care)	
Study 2: health knowledge: data obtained from stroke rehabilitation team members and chart audits		
Rehabilitation team member practice change	Chart audits will be conducted 6 months (+/−1 month) before CO-OP KT implementation as a baseline and to confirm practice gaps previously identified with interviews [4], and repeated at 6 and 12 months (+/−1 month) following the CO-OP KT intervention. The chart audit review criteria will center around documentation of functional goals (e.g., independence with upper body dressing), rather than impairment-reduction goals (e.g., increase arm strength); evidence of patient involvement in the goal-setting process; evidence of teaching of cognitive and problem-solving strategies as an intervention technique; evidence of use of guided discovery as an intervention technique	• −T6 • T6, T12
Stroke rehab professional self-efficacy with knowledge and skills related to CO-OP	CO-OP essential elements self-efficacy tool: participants are asked to rate their ability to perform 25 elements on a 10-point scale, with 1 indicating that they cannot perform the element at all and 10 indicating that their performance is excellent. Face validity evaluated by five members of the International CO-OP Academy	• −T1 • T1 • T12
Team perceptions and experiences with team processes, practices, attitudes related to adoption and sustainability of best practices for cognitive rehabilitation	Semi-structured site-specific focus group with groups of 5–8 team members at a time. Focus groups will be conducted by experienced facilitator Dr. Anne Hunt who will begin with an open-ended question “What has been your experience with facilitating recovery in patients with cognitive impairment?” Based on responses, Dr. Hunt will probe to obtain a thorough understanding of perceptions and experiences from a wide range of team members at each site	• −T1 • T12
Study 3: health outcomes: data obtained from consenting individual patients		
Performance on personally-meaningful, self-selected activities	The Canadian occupational performance measure (COPM) is a standardized instrument for eliciting performance issues from the client perspective, and for capturing perceived changes in performance over time [16]. The COPM has demonstrated test-retest reliability of 0.89 in people with stroke [34]. A change of 2 points or more on the COPM is considered clinically significant [16]	• Admission to inpatient rehabilitation • Discharge from inpatient rehabilitation • 1 month post discharge • 3 months post discharge • 6 months post discharge
Self-efficacy to perform daily activities	The self-efficacy gauge (SEG) was designed to measure an individual’s self-efficacy in his or her ability to perform daily occupations that span a range of self-care, productivity, and leisure activities. Participants are asked to rate their confidence in their ability to perform 28 items, each on a 10-point scale, with 1 representing “not confident at all” and 10 representing “completely confident”. The SEG has very high internal consistency (0.94) and test-retest reliability (0.90) [35]	
Health status	The stroke impact scale (SIS) [36] is a 59-item questionnaire about the perceived impact of stroke on function and everyday life. The SIS evaluates eight domains. Each item is scored on a 5-point Likert scale related to the degree of difficulty the person with stroke is experiencing. The SIS is widely used in stroke intervention studies as an outcome measure and the psychometric properties of the instrument are well defined [36–38]	
Cognitive screening tool	The MoCA is a 30-item test of cognitive impairment that includes elements of short-term memory recall; visuospatial capacity; aspects of executive functioning; attention, concentration, and working memory; language; and orientation [29]. The MoCA has excellent internal consistency (0.83) and test-retest reliability (0.92) [29]	

*CO-OP* cognitive orientation to daily occupational performance, *KT* knowledge transfer

### Study 3: patient activity, participation, and self-efficacy outcomes

To answer question 3, a non-randomized study using historical controls will be conducted. We will compare a group of patients treated by stroke teams who have been exposed to the CO-OP KT intervention (intervention) to a group who have not been exposed (historical control). Prior to the implementation of CO-OP KT, control participants will be recruited, and after the CO-OP KT intervention, intervention participants will be recruited. During the 3-month period when the CO-OP KT intervention is being administered to teams and learning is being consolidated, no recruitment will occur. In addition to data elements universally collected for the ITS Study 1, Study 3 participants will undergo assessments to measure activity performance, participation, and self-efficacy.

Data collection points will occur at the individual patient participants’ admission to rehabilitation, discharge from rehabilitation, and at 1, 3, and 6 months following discharge. Follow-up assessments will be administered by telephone, a feasible and cost-effective alternative to face-to-face assessments [27]. Data will be collected by family member proxy if the patient participant is not capable of a telephone interview [28]. Table 1 provides a description of all outcomes, indicators, and timing of administration.

### Recruitment and sample sizes

Five inpatient stroke rehabilitation units or combined stroke/neurology units in the TSNs have agreed to participate. Based on past experience, we estimate that the five units together will have approximately 80 admissions per month combined to contribute to the aggregate monthly data. For Study 1 (admission to rehabilitation), we will collect data from all patients aged 18 years or more who have completed inpatient rehabilitation with a primary diagnosis of stroke, defined as Rehabilitation Care Group (RCG) 01.1, 01.2, 01.3, 01.4 or 01.9. For Study 3, a subset of those patients will be recruited and the additional criteria of having at least some cognitive impairment will be applied, determined using the Montreal Cognitive Assessment© (MoCA©) [29]. The MoCA© is a 30-item test of cognitive impairment that includes elements of short-term memory recall; visuospatial capacity; aspects of executive functioning; attention, concentration, and working memory; language; and orientation. Patients with scores lower than 26 will be included. Additionally, patients will be required to have sufficient English language skills to complete the study assessments (see Table 1 for descriptions). Exclusion criteria are neurological diagnoses other than stroke, presence of major psychiatric illness or capacity issues requiring the use of a substitute decision maker under the Ontario substitute decision maker act. Based on data from a published acute rehabilitation CO-OP study [14], a sample size of 13 per group will have 80 % power to detect a between-group difference of 9 points on the FIM™, standard deviation of 8. Allowing for 30 % attrition from all sources, we will recruit 17 participants per group, 34 in total. Based on past projects, we expect a consent rate of one participant per site per month. Thus, recruitment is highly feasible, and will likely be completed in 4 months for each group.

For Study 2 (changes in team members practice, knowledge, and self-efficacy), all clinicians will be invited to participate, and those who provide informed consent will be included in the survey and focus groups. The five stroke teams have approximately 50 staff each, or approximately 250 total. Based on past experience, we expect a participation rate of 50 % for the self-efficacy survey and about 30 % for the focus groups. Therefore, we anticipate running 2 focus groups (1 pre-intervention and 1 post-intervention) with 8–10 participants each at each of the 5 sites, for a total of 10 focus groups. Chart audits will be conducted on 80 charts of patients discharged approximately 6 months (±1 month) before CO-OP KT implementation, and at 6 and 12 months (±1 month) after CO-OP KT implementation, 240 charts total, providing 89 % power to determine a 25 % change in the audit criteria outlined in Table 1 [30].

### Data analysis

Descriptive statistics will be compiled for all quantitative data collected. The interrupted time series (Study 1) will be analyzed using simulation modeling analysis [31], a trend analysis that provides good power to detect post-intervention trend changes when analyzing 30 time points or less per segment. As a precursor to modeling, the time series data will be plotted, analyzed visually for trends, and the degree of autocorrelation will be calculated. For Study 2, pre/post-changes on the clinician self-efficacy survey will be calculated using the paired *t* test, assuming the data are normally distributed or the signed rank test if not. Qualitative focus group data will be analyzed using directed content analysis [32], with initial codes based on questions derived from the focus group questions.

For Study 3, the non-randomized controlled trial of individual patients, between- and within-group differences on the outcome measures will be examined using repeated measures ANOVA.

### Description of the CO-OP KT intervention

CO-OP KT consists of CO-OP training for the inter-professional team and subsequent implementation support (KT). Two levels of CO-OP training will be provided: (1) A 1 to 2 hour introduction to CO-OP, using an e-learning module and (2) advanced training in the form of a 3-day hands-on CO-OP workshop. Team members (e.g., occupational therapists, physiotherapists, speech-language pathologists, etc.) who are directly involved with teaching the skills for which CO-OP is effective, such as mobility, activities of daily living, or communication, will receive both training components. Team members who are not directly involved in teaching functional skills (e.g., physicians, most nurses, etc.) will be introduced to CO-OP using the first introductory e-learning module so that they can support and encourage the use of the approach. Funds have been budgeted to cover clinician time when they are participating in either level of training.

**Fig. 3:**
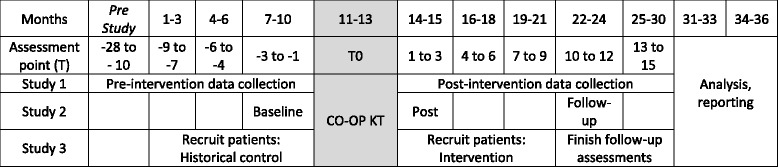
Project design and timeline

KT support based on the Graham et al.’s knowledge to action framework will be provided to facilitate implementation and sustainability of the CO-OP approach [20]. The KT support uses local-adapted knowledge and evidence-based behavior change strategies and will be delivered by an implementation facilitator, to be employed by the project. We will recruit an implementation facilitator who is a health-care professional with experience in both stroke rehabilitation and in implementing educational and knowledge translation initiatives. He or she will make linkages between CO-OP content and existing TSNs KT infrastructure. See Fig. 3 for a detailed visual depiction of existing infrastructure. The implementation facilitator will work closely with project knowledge users MD (regional education coordinator), SQ (regional rehab & community re-engagement coordinator), and SR (clinical nurse specialist). Content will be tailored for the unique environment of each individual team, and the implementation facilitator will be available throughout the project for consultation by telephone or email, will moderate a CO-OP discussion forum on the TSN’s online virtual community of practice (VCoP) website (www.strokecommunity.ca), and will be onsite at each participating institution regularly throughout the project for one-on-one face-to-face coaching and issue resolution. These latter measures will be enacted to meet the knowledge to action framework’s requirements of monitoring and sustaining knowledge use.

### Sustainability

Both the sustainability of training and the sustainability of knowledge uptake have been considered. The project has formed a partnership with a group at an educational institution who will develop, implement, and monitor a CO-OP e-learning module, ensuring it will be available and updated on a long-term basis. The e-learning module will include both the 1–2 h introduction to CO-OP and the multi-hour CO-OP workshop. It will provide clinicians who join the inter-professional teams after the CO-OP KT implementation with a means of receiving advanced CO-OP training in a timely and cost-effective manner. Sustainability of uptake will be ensured by Toronto Stroke Networks’ KT infrastructure currently in place, as well as through sustainability processes developed locally at each site, in collaboration with the implementation facilitator.

### Potential challenges and their solutions

An issue inherent to time series studies is the possibility that changes not related to the intervention will occur within the system and impact the outcome of interest. By collecting pre-intervention data for 28 months, these trends will be apparent and can be taken into account during analysis. Additionally, time series data will be examined in two ways: we will examine all five units as a group, and also as individual cases. By examining the trends of individual units, any outcome-impacting changes within a single unit, such as a sudden staffing shortage, can be detected and explained.

Influencing practice changes among an entire health system is expected to be challenging. In addition to addressing knowledge, skills, and attitudes of individual clinicians, institutional culture and support for practice change will be significant factors. The implementation facilitator will work together with teams to develop local, site-specific content to help mitigate some of these concerns. Two pragmatic barriers to evidence uptake by health-care professionals are lack of time and lack of resources. We have budgeted for clinician time to attend workshops. Additionally, we have included the position of the implementation facilitator to provide the teams with a human resource with the time and ability to develop materials on their behalf and to provide support while new knowledge and skills are being adopted and consolidated. The use of the VCoP across sites also provides just-in-time access to peers and experts to support ongoing learning and implementation needs. Access to rehabilitation for persons with cognitive impairment has been identified as a priority by the TSNs acute and rehab stroke flow task groups, with representation from decision-makers across Toronto organizations. These decision-makers have made a collective commitment to local implementation and to supporting this project across the system.

Patient recruitment among the stroke patient population is challenging [33]. To mitigate this and reduce the burden on point of care clinicians, our inclusion criteria are broad and we have dedicated research assistant time for recruitment and retention.

The implementation facilitator will monitor CO-OP intervention fidelity to ensure that the rehabilitation team members are adopting CO-OP appropriately. He or she will observe at least one video recorded treatment session from each team member who has taken the advanced CO-OP workshops and rate their use of CO-OP using an existing CO-OP treatment fidelity checklist (http://ot.utoronto.ca/coop). This will also act as a feedback and instructional mechanism. The implementation facilitator will keep a journal of all implementation activities, successes, and challenges as the project unfolds. As well, regular reporting on the local status of CO-OP implementation will occur to Toronto Stroke Networks decision-makers through existing channels.

### Trial status

Not yet recruiting.

### Ethics

The study received lead site Research Ethics Board approval from the Sunnybrook Health Sciences Centre Human Research Protections Program (PIN 177-2015).

## Discussion

This project will advance general knowledge about the degree to which the implementation of a supported, integrated, inter-professional KT initiative can sustainably change health system outcomes (access to rehabilitation), knowledge outcomes (rehabilitation team practice), and patient outcomes (functional improvement). Additionally, it will advance knowledge about the degree to which changes can be sustained over the longer term through integrated KT mechanisms and committed partnerships with health and academic institutions.
